# The inhibitory effect of KIAA1456 on the proliferation and metastasis of epithelial ovarian cancer through SSX1 and AKT signaling pathway

**DOI:** 10.7150/jca.81587

**Published:** 2023-03-27

**Authors:** Yingfeng Zhang, Congcong Sun, Yanhong Gao, Yanhua Mao, Benyuan Wu, Changjiang Li, Wenwen Zhang, Jia Wang

**Affiliations:** 1 University-Town Hospital of Chongqing Medical University, Chongqing, China, 401331; 2 Fuling Central Hospital of Chongqing, Chongqing, China, 400000

**Keywords:** Akt signaling pathway, epithelial ovarian cancer, KIAA1456, SSX1, proliferation

## Abstract

**Background:** KIAA1456 is effective in the inhibition of tumorigenesis. We previously confirmed that KIAA1456 inhibits cell proliferation and metastasis in epithelial ovarian cancer (EOC). In the current study, the specific molecular mechanisms and clinical significance of KIAA1456 underlying the repression of EOC were investigated.

**Methods:** Immunohistochemistry was used to evaluate the protein expression of KIAA1456 and SSX1 in EOC and normal ovarian tissues. The relationship of KIAA1456 and SSX1 with overall survival of patients with EOC was analysed with Kaplan-Meier survival curve and log-rank tests. KIAA1456 was overexpressed and silenced in HO8910PM cells with lentivirus. Anticancer activities of KIAA1456 was tested by CCK8, plate clone formation assay, flow cytometry, wound healing assay and Transwell invasion assay. Xenograft tumour models were used to investigate the effects of KIAA1456 on tumour growth *in vivo*. Bioinformatics analyses of microarray profiling indicated that SSX1 and the PI3K/AKT were differentially expressed in KIAA1456-overexpressing and control cells. The downstream factors of PI3K/AKT that are related to cell growth and apoptosis.

**Results:** KIAA1456 expression was lower in EOC than in normal ovarian tissues. Its expression negatively correlated with pathological grade. Pearson's correlation analysis showed that KIAA1456 negatively correlated with SSX1 expression. The overexpression of KIAA1456 in HO8910PM cells inhibited proliferation, migration and invasion and promoted apoptosis. The silencing of KIAA1456 resulted in the opposite behaviour. A xenograft tumour experiment showed that KIAA1456 overexpression inhibited tumour growth* in vivo*. The overexpression of KIAA1456 inhibited SSX1 and AKT phosphorylation in HO8910PM cells, causing the inactivation of the AKT pathway and eventually reducing the expression of PCNA, CyclinD1, MMP9 and Bcl2. The silencing of KIAA1456 resulted in the opposite behaviour. SSX1 overexpression could partially reverse the KIAA1456-induced biological effect.

**Conclusion:** KIAA1456 may serve as a tumour suppressor via the inactivation of SSX1 and the AKT pathway, providing a promising therapeutic target for EOC.

## Introduction

Ovarian cancer is one of the most common malignancy worldwide and the most lethal gynaecological malignancy because of diagnosing at an advanced stage and poor prognosis 1.The share of ovarian cancer deaths in Asia and Africa are higher than other regions because of different distribution of cancer types, the increased prevalence of smoking, the westernized dietary patterns and obesity, etc. 2. Ovarian cancer encompasses a heterogenous group of malignancies that vary in origin, pathologic grade, and numerous other characteristics. Ninety percent of ovarian cancers are epithelial in all racial/ethnic groups, which are further grouped as type I (low grade) or type II (high grade) based on clinicopathologic factors 3. To date, a variety of approaches, such as functional genomics, systems biology and proteomics, have been used to develop different methods for the early and specific detection of ovarian cancer 4,5. In our previous research, we confirmed that KIAA1456 (human tRNA methyltransferase 9-like [hTRM9L]) inhibits the proliferation and metastasis of EOC cells 6.

In recent years, it has been demonstrated that KIAA1456 has an anticancer effect in tumors. KIAA1456, also known as TRM9L (transfer RNA (tRNA) methyltransferase9-like gene), which located on chromosome position 8p22 7. Mammalian TRM9L, in company with its paralog, AlkB homolog 8 (ALKBH8), are signed homologs of the Saccharomyces cerevisiae tRNA methyltransferase 9 (Trm9) enzyme 7,8. The proteins coded by ALKBH8 and Trm9 are enzymes that catalyze the swing of tRNA, playing important roles in oxygen pressure regulation, DNA damage repair and cell pressure signal regulation in tumor cells 9,10. Normal protein translation and DNA damage repair can be disrupted by the abnormal expression of KIAA1456, which ultimately leads to the cancerization and malignant proliferation of cells 11. Prior studies showed that KIAA1456 expression level in adult brain tissue was the highest and relatively low level in skeletal muscles, testicles, and ovaries 12. Simultaneously, KIAA1456 expression is greatly reduced or silenced in breast, cervical, colorectal, bladder and testicular carcinomas via epigenetic mechanisms 11,13. In further support of a tumour inhibition role for KIAA1456, our previous study confirmed that KIAA1456 inhibits the proliferation and invasion of EOC cells, highlighting the need for a clearer understanding of the mechanism and pathways involved.

Chromosomal translocations are a primary source of genetic aberrations that cause certain malignancies. The genes of synovial sarcoma X (SSX) breakpoint multigene family were originally recognised as fusion partners to the SYT gene in synovial sarcomas harbouring the t(X;18) translocation 14. The chromosomal translocation t(X;18)(p11.2;q11.2) contributes to the fusion of the SS18 (also known as SYT) gene on chromosome 18 to the SSX1, SSX2 or SSX4 gene on the X chromosome 15. The SSX1 to SSX9 genes have been identified in previous studies, they are localised in the nucleus and interact with a variety of proteins 16. The synovial sarcoma X breakpoint antigen 1 (SSX-1), along with other members of the SSX family frequently expressed in cancer, has been verified to be an oncoprotein involved in disease pathogenesis and a molecular therapeutic target against synovial sarcoma; hence, it may be an excellent candidate target for cancer immunotherapy 17. In general, the expression and activity of the SSX antigens are promising targets for the specific immunotherapy of cancer. The oncogene SS18-SSX1 facilitates tumourigenesis by increasing the expression of SHCBP1 and lowering the PI3K/AKT/mTOR and MAPK/ERK signalling pathways and cyclin D1 expression; hence, SS18-SSX1 generally acts as a tumour promoter 18.

The PI3K/AKT signalling pathway is a major signalling pathway associated with cancer proliferation and metastasis at the genetic level, leading to the activation and recruitment of the AKT serine/threonine kinase 19,20. All kinds of downstream protein substrates, such as death promoter of Bcl-2 and GSK3b, can be phosphorylated while AKT be activated by phosphorylation 21. An abnormality in the PI3K/Akt/mTOR signalling pathway can lead to cell cycle advancement, tumourigenesis, G1 and G2 cycle arrest impairment, apoptosis inhibition and angiogenesis 20-24. The pivotal role of the PI3K/AKT pathway in the regulation of the proliferation, survival and invasiveness of ovarian carcinoma makes it a potential target for therapeutic intervention 21.

In the present research, we explored the expression of KIAA1456 in epithelial ovarian tumour specimens and EOC cell lines and ascertained the relationships between KIAA1456 expression and the clinicopathologic characteristics of patients with EOC. In addition, the effect of KIAA1456 on proliferation and metastasis was tested after overexpressing and down-expressing this gene in EOC cells. Grounded on our findings, we demonstrated that KIAA1456 inhibits EOC cell proliferation, migration and invasion and promotes apoptosis by suppressing SSX1, thereby inactivating AKT signalling and its downstream targets. Altogether, this evidence clarifies that KIAA1456 plays an important role in the development and progression of EOC and may serve as a possible therapeutic target for epithelial ovarian tumours.

## Materials and methods

### Patients and tissue preparation

A total of 45 epithelial malignant cancer specimens, 15 benign epithelial ovarian tumour specimens, and 15 normal ovarian tissues were acquired between 2012 and 2017 in the First and Fourth Affiliated Hospitals of Chongqing Medical University. The eligibility criteria were as follows: (1) Epithelial ovarian cancer (EOC), all specimens were authorized with pathological diagnoses. (2) Stage was assigned based on the operative findings and histopathological results according to the 2014 FIGO staging system for ovarian cancer. Patients were excluded from analysis if they (1) had previous history of another malignancy, (2) received neoadjuvant chemotherapy as a primary treatment, (3) were diagnosed with multiple primary malignancies before or during cytoreductive surgery, (4) suffered recurrent EOC, (5) if they had incomplete preoperative laboratory data or did not consent to the use of their medical records for research. Controls (Patients who have had their ovaries removed due to benign epithelial ovarian tumours and other benign uterine diseases) had the same eligibility criteria as the cases. The clinical characteristics of patients were gathered, for instance, age and grade. The consent and approval of the patients were acquired for research purposes from the Institutional Research Ethics Committee of Chongqing Medical University.

### Cell culture and reagents

HO8910 and HO8910PM cell lines and the packaged retrovirus containing the KIAA1456 expression plasmid used in this study were produced in our previous work. The EOC cell lines were placed in RPMI 1640 complemented with 100 U/ml penicillin, 100 µg/ml streptomycin (Hyclone) and 10% FBS and incubated in 37°C with 5% CO_2_ atmosphere. Antibodies against KIAA1456 and SSX1 were purchased from Abcam, and phospho-AKT, AKT, PCNA, Bcl-2, MMP9 and CyclinD1 were procured from Cell Signaling Technology (Danvers, MA, USA). β-actin and GAPDH were obtained from Beyotime (China).

### Immunohistochemistry

Paraffin-embedded tissue sections were deparaffinised with xylene and rehydrated with graded ethanol. Antigen retrieval was accomplished by incubating the specimens with boiled citrate buffer for 5 min at 100°C. Hydrogen peroxide (3%) and serum were used to block endogenous peroxidase activity and non-specific antigens. The specimens were then incubated with KIAA1456(1:100) and SSX1(1:100) antibodies overnight at 4°C. Afterwards, the specimens were incubated with goat anti-rabbit secondary antibody (1:500). After each treatment, the specimens were developed using 3,3-diaminobenzidine solution and counterstained with hematoxylin. After staining, the slides were critiqued by two independent observers using a microscope (DM6000B). The immunohistochemical staining of KIAA1456 and SSX1 was computed based on the population of positive cells and staining intensity. Positive expression (The staining scores were calculated by both percentage of positive cells and color intensity) was graded as follows: <10% (negative staining), 10%-20% (weak staining), 20%-30% (moderate staining), >30% (strong staining).

### Lentivirus transfection

KIAA1456 lentivirus (LV- KIAA1456) used for KIAA1456 overexpression, SSX1 lentivirus (LV-KIAA1456-SSX1) used for SSX1 overexpression, both lentiviral constructs expressing KIAA1456 shRNA (RNAi- KIAA1456), matching control lentivirus (shcon) (LV-NC, RNAi-NC and LV-KIAA1456-NC) and negative control lentivirus (con)(HO8910PM and LV-KIAA1456) were obtained from Genechem Co. Ltd (Shanghai, China). For transfections, HO8910PM and LV-KIAA1456 cells were cultured in media to probably 50% clustering in six-well plates. These lentiviruses were introduced into HO8910PM or LV-KIAA1456 cells with 8 µg/ml polybrene (Genechem) and complete medium. A fluorescence microscope was applied to detect the transfection effects after 48 h. The transfected cells were purified with puromycin. The effective forced expression of KIAA1456 and SSX1 was tested by real-time PCR and Western blot analysis after 72 h.

### Quantitative real-time PCR

RNAiso Plus (TaKaRa, Beijing, China) was used to extract total RNA in cells and tissues. The Reverse Transcription Kit (Takara, Dalian, China) was used to reverse-transcribe RNA into cDNA. Real-time PCR analyses were performed using Power SYBR Green (Takara, Dalian, China). The results were standardised to the expression level of GAPDH. The relative fold-changes of mRNA levels were counted according to the 2 - ΔΔCT method. All qRT-PCR experiments were done in triplicate. Student's t-test was used to compare differences in gene expression levels between groups. A *p* value < 0.05 was considered to indicate a significant difference. Primers were synthesised by Shanghai Sangon Biological Engineering Technology.

### Western blot analysis

The tissues and cells were lysed by RIPA Lysis Buffer (Beyotime, China) containing PMSF and a phosphatase inhibitor. The BCA protein assay kit (Beyotime, China) was used to measure protein concentration. Cellular protein lysates were separated via SDS-PAGE and transferred to PVDF membranes using a standard protocol. The membranes were incubated with primary antibodies (including KIAA1456(1:500), SSX1(1:500), AKT (1:1000), p-AKT (1:2000), PCNA (1:1000), Bcl2(1:500), CyclinD1(1:1000) and MMP9(1:500)) overnight at 4°C after blocking with 5% non-fat milk. Subsequently, the membranes were incubated with the *the appropriate* secondary antibodies. HRP-conjugated anti-mouse/rabbit IgG (1:5000) was applied to the secondary antibody. Protein bands were detected using an enhanced chemiluminescence system. GAPDH (1:2000) and β-actin (1:1000) were used as loading controls. Digital images were quantified using the Quantity-One software (Bio-Rad, USA).

### Cell viability assay

To detect cell growth ability, we cultivated cells with a density of 2000 cells/well into 96-well plates and incubated them at 37 °C. For 24, 48, 72 and 96 h; cells in each well were added with 10 μl of Cell Counting Kit-8 (Beyotime) and then incubated for 1 h at 37°C. Absorbance values were read at 450 nm employing an enzyme-labelled instrument. Data were analyzed using GraphPad Prism 5.0. The assay wasperformed three times in triplicates, and the data presented as mean ± SD.

### Plate colony formation assay

For a plate colony formation assay, the same amounts of different cells were cultivated in a six-well plate (500 cells/well) for 10-14 days. The plate was replenished with fresh medium every 3 days. Finally, the cells were fixed with 4% paraformaldehyde and stained with freshly prepared crystal violet for 20 min. Colony formation was assessed under a microscope (Nikon, Japan).

### Cell cycle and apoptosis analysis with flow cytometric assay

HO8910PM, LV-KIAA1456, LV-SSX1, RNAi-KIAA1456 and their control cells were seeded in a six-well plate (5×10^5^ cells/ well). Cells were harvested after 24 h of incubation, washed with PBS and then fixed in 70% ice-cold ethanol overnight at -20°C. Cells were cultivated at 37 °C for 30 min with 10 mg/ml RNase (Sigma, St. Louis, MO, USA) and 50 mg/ml propidium iodide (Sigma). Flow cytometry (BD Bioscience, San Jose, CA, USA) was used to analyse the cell cycle. Conforming to the manufacturer's protocol, reactants from the Annexin V-FITC apoptosis kit (BioVision, Wuhan, China) were used to cultivate and incubate the same treated cells to test for cell apoptosis. We conducted three independent experiments to determine the Cell cycle and apoptosis, and the data indicated as mean ± SD.

### Immunofluorescence

Cells were attached to slides, fixed with 4% paraformaldehyde, incubated in 0.3% Triton X-100 for 15 min and then blocked with 5% goat serum. The cells were then incubated with primary antibodies against KIAA1456 and SSX1 at 4°C overnight and then with suitable secondary antibodies. DAPI (Beyotime) was used to counterstain the nuclei. Immunofluorescent signals were measured using a microscope (DM6000B).

### Wound-healing cell migration analysis

Different cells with a concentration of 6×10^5^ cells/well were seeded into six-well plates filled with complete RPMI 1640 medium, which then formed a confluent monolayer. Thereafter, a 200 μl pipette tip was used to scratch the plate to form perpendicular wounds in the monolayers. Floating cells were washed with PBS and continuously incubated in complete medium. At 0, 24, and 48 h after wounding, scratched areas were photographed with a microscope, and the width of the wound was measured using the Image J software. All assays were performed in triplicate.

### Transwell invasion assays

The invasion ability of LV-KIAA1456, RNAi-KIAA1456 and LV-SSX1 cells was investigated with 6.5 mm chambers with 8 μm pores (Corning, USA). The same amounts of cells were plated on the top side of polycarbonate Transwell filters lidded with 1 μg of Matrigel in the upper chambers with serum-free RPMI 1640 medium (5×10^4^ cells). The bottom chambers were appended with 600 μl of RPMI 1640 medium complemented with 10% FBS. Invaded cells were fixed with methanol and dyed with hematoxylin and eosin after 12 h of incubation. Images of each membrane were captured in five random fields (×100 magnification) in a microscope. Statistics were done using GraphPad Software.

### Xenograft tumor model

The female nude mice (4 weeks old) used in this research were supplied by the experimental animal centre of Chongqing Medical University. All animal experiments were authorised by the Ethics Committee of Chongqing Medical University. The LV-KIAA1456 and control cells at a density of 2×10^6^ cells per 50 μl were re-suspended in RPMI 1640 and injected subcutaneously into the nude mice. The tumours were measured when they were grown out every 7 days, and the volume was measured by the formula length × width^2^ ×1/2. The nude mice were sacrificed after 35 days of inoculation. Tumour tissues were resected, weighed and then used for IHC.

### Statistical analysis

Statistical analyses were implemented using SPSS 22 and GraphPad Prism 5.0 software. Differences between the two groups were evaluated by Student t-test. One-way ANOVA was used when comparing multiple groups. Clinical data were analyzed using the chi-square test. Association between KIAA1456 and SSX1 expression in epithelial ovarian cancer tissue was assessed using Spearman's rank correlation test. The prognostic significance analysis was executed using Kaplan-Meier analysis and log-rank tests. Data were represented as mean ± S.D. of at least three independent experiments. The criteria of statistical significance was P<0.05.

## Results

### KIAA1456 is lowly expressed in human epithelial ovarian cancer tissues

**Figure 1:**
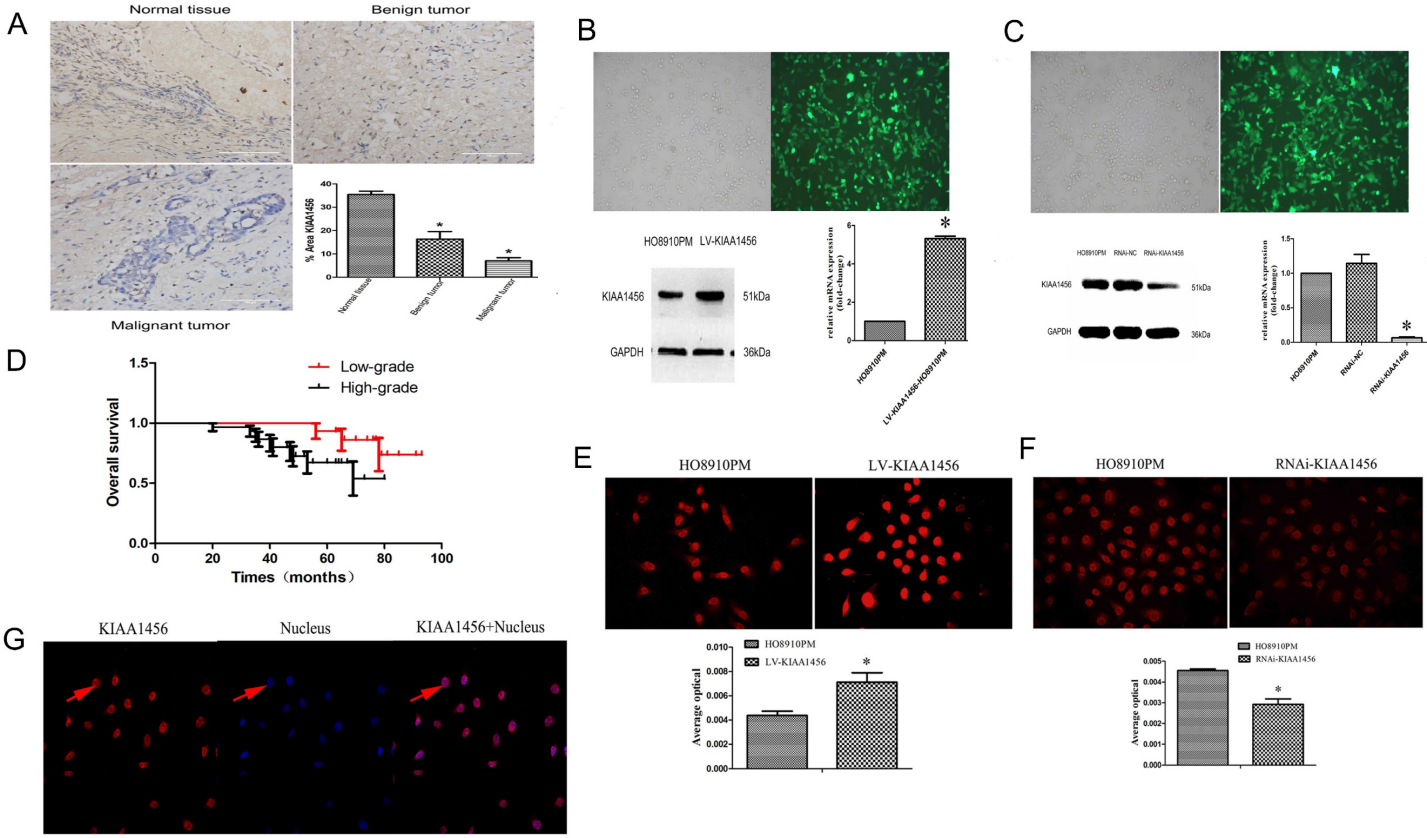
KIAA1456 was expressed in the nucleus of the EOC cells. Low KIAA1456 expression is predictive of poor prognosis for patients with epithelial ovarian cancer. (A) Representative images of KIAA1456 immunostaining in normal ovarian tissues, benign epithelial ovarian tumor tissues and malignant epithelial ovarian cancer tissues. The scale bars indicate 100 μm in the inserts. The results of KIAA1456 expression were evaluated by the staining scores. *p < 0.05. (D) Kaplan-Meier analysis of overall survival based on KIAA1456 expression in 45 patients with EOC, statistically significant differences were observed between groups (P<0.05). (G) KIAA1456 in HO8910PM cells expressed in the nucleus which was detected by laser confocal. (B, E) KIAA1456 was overexpressed in HO8910PM cells by lentivirus transfection. Protein and mRNA of KIAA1456 were detected by Western blot analysis, RT-PCR and immunofluorescence (200×). (C, F) KIAA1456 was silenced in HO8910PM cells by lentivirus transfection. Protein and mRNA of KIAA1456 were detected by Western blot analysis, RT-PCR and immunofluorescence (200×). GAPDH was used as a loading control. The data was statistically significant at *p < 0.05 as compared to negative control. The data correspond to the mean ± SD of three independent experiments.

We confirmed that KIAA1456 has lower expression in HO8910PM cells than in HO8910 cells. To evaluate the protein expression levels of KIAA1456 in EOC tissues, we performed immunohistochemistry (IHC) to analyse 45 malignant EOC specimens, 15 benign epithelial ovarian tumour specimens and 15 normal ovarian tissues. The data confirmed that the protein expression of KIAA1456 in epithelial ovarian cancer specimens was notably lower than that in both normal ovarian tissues and benign tumour tissues (Figure 1 A). Then, the relationship between KIAA1456 expression and pathological grade of EOC tissues was analysed. We found that the expression levels of KIAA1456 significantly correlated with the pathological grade. In contrast with low-grade EOCs, high-grade EOCs showed low KIAA1456 expression; furthermore, no difference was found in the expression of KIAA1456 in age (Table SI). In addition, a Kaplan-Meier survival curve and log-rank tests showed that patients with ovarian carcinoma with less KIAA1456 expression have a close relationship with poor overall survival (Figure 1 D).

### KIAA1456 suppresses proliferation, invasion and migration of epithelial ovarian cancer cells

HO8910 and HO8910PM EOC cell lines were chosen for succeeding experiments after mRNA and protein exploration in our previous work. Laser confocal analysis was implemented to identify the location of KIAA1456 in HO8910PM cells, and the results showed that KIAA1456 was expressed in the nucleus (Figure 1 G). To test the anticancer activity of KIAA1456 in EOC cells, we retrovirally established a stable overexpression of KIAA1456 in HO8910PM cells (designated as LV-KIAA1456) and silenced KIAA1456 in HO8910PM cells (designated as RNAi-KIAA1456) and control cells, as described in the Materials and Methods section. The levels of KIAA1456 mRNA and protein in LV-KIAA1456 (Figure 1 B,E) and RNAi-KIAA1456 cells (Figure 1 C,F) were detected by qRT-PCR, Western blot and immunofluorescence.

**Figure 2:**
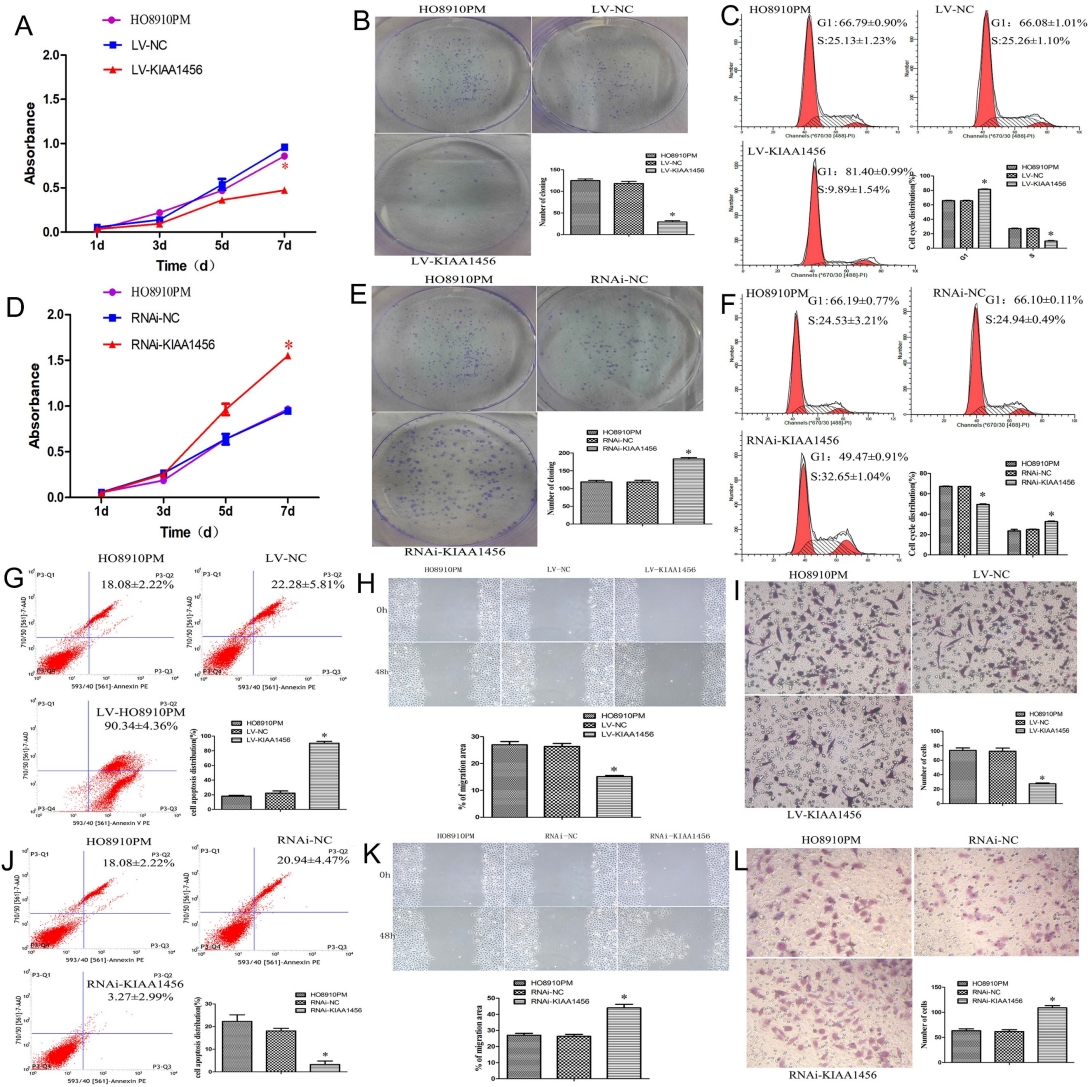
The upregulation of KIAA1456 suppresses proliferation, invasion and migration and promotes the apoptosis of epithelial ovarian cancer cells, whereas the silencing of KIAA1456 in HO8910PM cells induces the opposite reaction. Cell viability and proliferation was detected in HO8910PM, LV-KIAA1456 and RNAi-KIAA1456 cells by CCK-8 (A and D) and colony formation (B and E) assays. Cell cycle distribution (C and F) and cellular apoptosis (G and J) were tested in HO8910PM, LV-KIAA1456 and RNAi-KIAA1456 cells by flow cytometry. HO8910PM, LV-KIAA1456 and RNAi-KIAA1456 cells were subjected to wound healing migration assay (H and K) and Transwell invasion assay (I and L). The data was statistically significant at *p < 0.05 as compared to control. Data are represented as mean ± SD of three independent experiments.

The effect of KIAA1456 on HO8910PM cell viability and growth were investigated by CCK8 assays and plate clone formation assay. LV-KIAA1456 cells grew significantly more slowly and generated fewer numbers and smaller colonies than the controls (Figure 2 A and B). However, KIAA1456 downregulation dramatically increased HO8910PM cell growth and clonogenicity (Figure 2 D and E). These data imply that KIAA1456 has a negative effect on the growth of human EOC cells.

Malignant tumours proliferate rapidly and derange normal cell cycle arrest to accomplish unrestrained cell proliferation. The abduction of cell cycle arrest is a crucial system and usual mechanism in a majority of malignant tumours, resulting in abnormal cellular proliferation. Flow cytometry assay was used to evaluate the effect of KIAA1456 overexpression on the cell cycle. The results revealed that the overexpression of KIAA1456 significantly increased the amount of G1 phase cells in LV-KIAA1456 cells and lessened S phase cells compared with control cells (Figure 2 C). By contrast, the downregulation of KIAA1456 reduced the population of G1 phase cells and augmented the percentages of the S phase (Figure 2 F). We investigated the effects of KIAA1456 on apoptosis in EOC cells. Apoptotic cells were markedly elevated in LV-KIAA1456 cells compared with the control cells (Figure 2 G). By comparison, the downregulation of KIAA1456 decreased the percentage of early and late apoptosis in EOC cells (Figure 2 J). Accordingly, these results demonstrated that KIAA1456 inhibits cell cycle progression and promotes the apoptosis of EOC cells.

Tumour metastasis and invasion are often involved in the reduction of cell-cell and/or cell-matrix adhesion. The effect of KIAA1456 on cell migration was first assessed by a wound healing assay. A significantly slower wound closure rate was observed in the KIAA1456 overexpressing cells compared with the controls at 48 h (Figure 2 H). Second, we performed a Transwell invasion assay in the EOC cell line. We found that LV-KIA1456 cells showed a minor degree of invasion through Matrigel (Figure 2 I). By contrast, silencing KIAA1456 dramatically increased the migratory and invasive capacity of HO8910PM cells (Figure 2 K and L). These results indicate that KIAA1456 inhibits migratory and invasive behaviours in EOC cells.

### KIAA1456 suppresses the growth of the epithelial ovarian cancer* in vivo*

**Figure 3:**
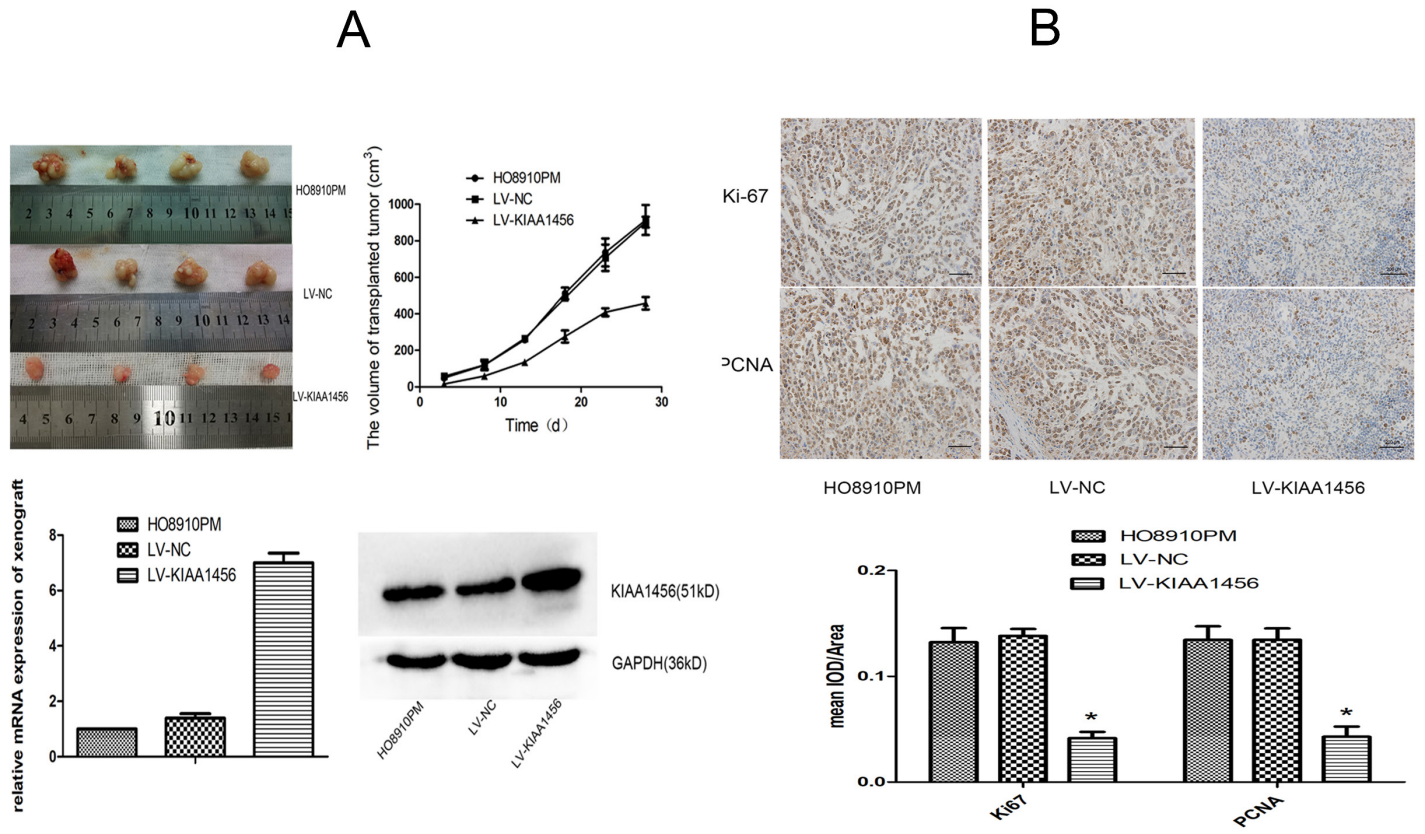
KIAA1456 suppresses the growth of epithelial ovarian cancer *in vivo*. (A) LV-KIAA1456 and corresponding control cells were subcutaneously injected into nude mice. (A and B) Growth curve and volume of xenografts in three groups. (C and D) Expression of KIAA1456 mRNA and protein in subcutaneous tumour xenografts in nude mice. (B) Immunohistochemical analysis of ki67 and PCNA levels in subcutaneous tumour xenografts (400×). Data represents the mean ± SD. *P <0.05.

To supplement our *in vitro* observations, we investigated the impacts of KIAA1456 on tumour growth* in vivo*. LV-KIAA1456 and matching control cells were subcutaneously injected into nude mice. As expected, the tumours from LV-KIAA1456 cells grew more slowly at the implantation location than the control group (Figure 3 A). The inhibited cell proliferation in the LV-KIAA1456-derived cancers was further corroborated by ki67 and PCNA levels (Figure 3 B). In summary, these results demonstrated that KIAA1456 has a negative effect on the proliferation of human EOC cells* in vivo*.

### Suppression of SSX1 is probably involved in the anticarcinogenic function of KIAA1456

To better understand the mechanisms by which KIAA1456 is involved in EOC development and progression, in our early work, we performed microarray analysis and identified a list of genes that were significantly differentially expressed after KIAA1456 overexpression, including the downregulation of SSX1 (Table SII). IHC was used to analyse the protein expression levels of SSX1 in the same EOC tissues. In contrast to KIAA1456, SSX1 was highly expressed in malignant EOC tissues relative to that in benign epithelial ovarian tumour tissues and normal ovarian tissues, and high-grade EOC presented a lower expression level of SSX1 than low-grade EOC (Figure 5 A). To determine whether KIAA1456 expression is correlated with SSX1 expression in EOC, we performed Pearson's correlation analysis between the expression levels of KIAA1456 and SSX1. The analysis showed that KIAA1456 was negatively correlated with SSX1 expression in EOC tissues (Figure 5 B).

**Figure 4:**
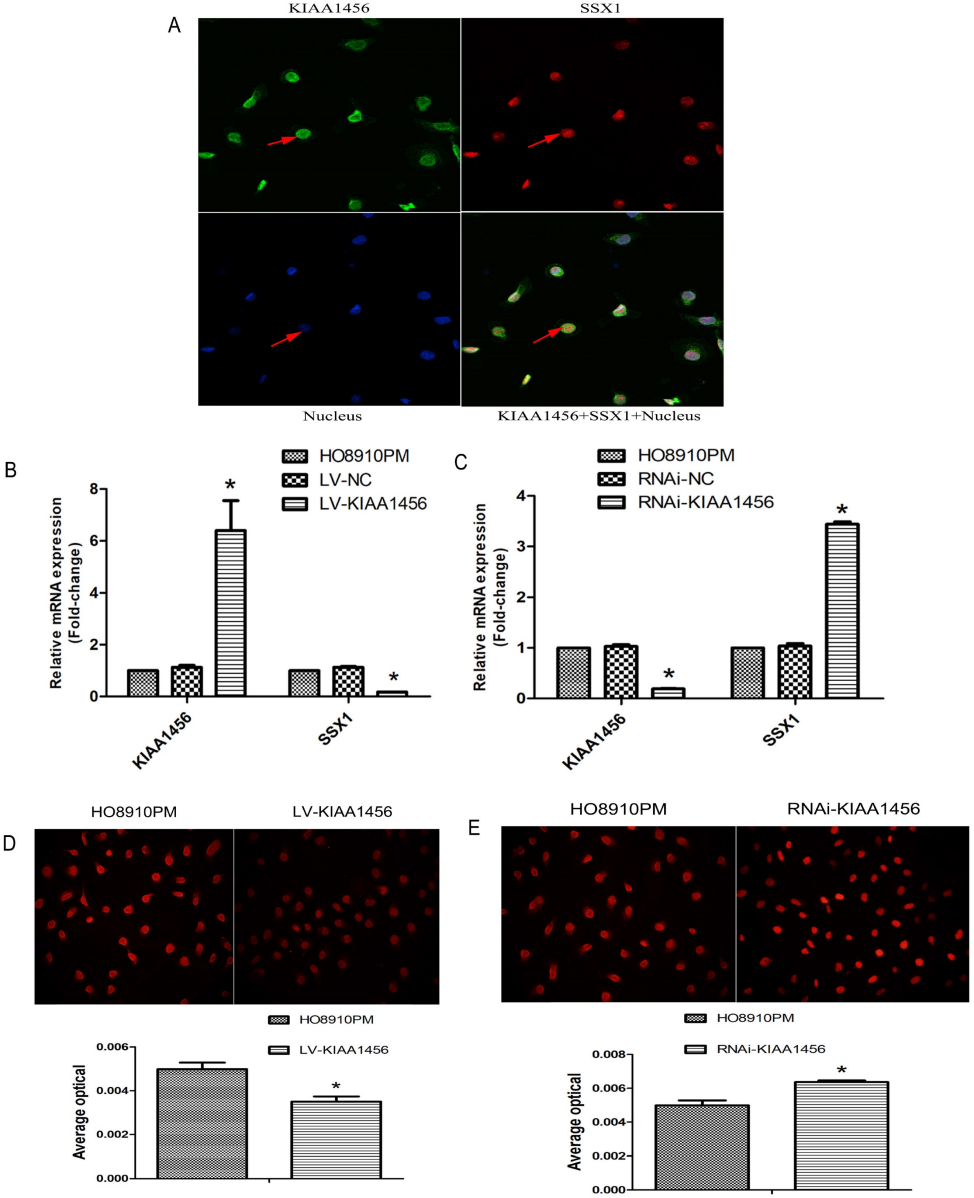
LV-KIAA1456 cells exhibited greatly decreased SSX1 protein and mRNA levels in EOC cells, whereas silencing KIAA1456 increased the protein and mRNA levels. (A) The laser confocal analysis showed that SSX1 was expressed in the nucleus. (B-E) The protein and mRNA of SSX1 were detected by RT-PCR and immunofluorescence (200×) in HO8910PM, LV-KIAA1456 and RNAi-KIAA1456 cells. Data were presented as mean ±S.D. of triplicates assays. *P <0.05.

**Figure 5:**
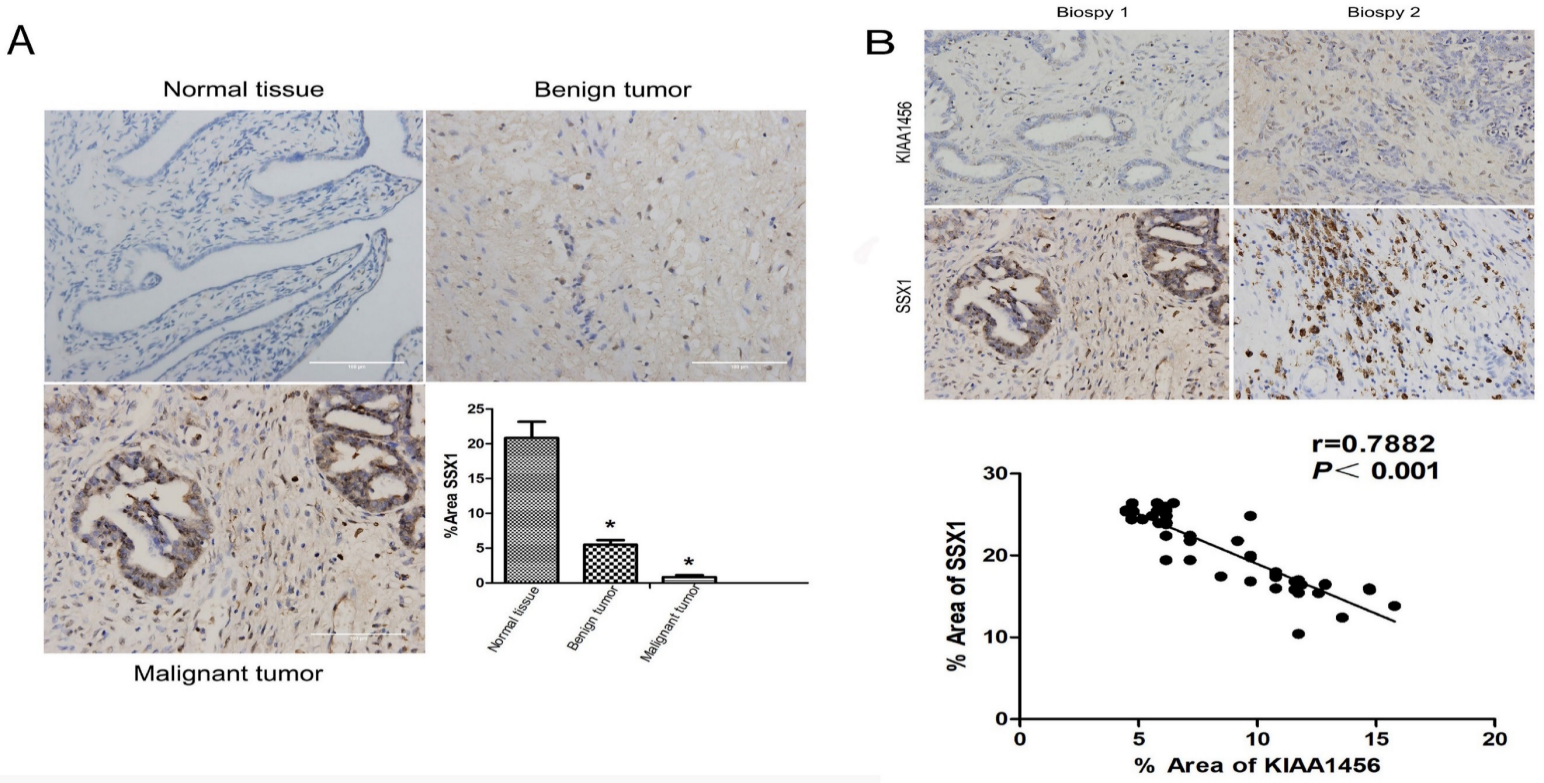
KIAA1456 was negatively correlated with SSX1 expression in EOC tissues. (A) Correlation of SSX1 expression in normal ovarian tissues and benign and malignant epithelial ovarian tumor tissues analysed using EOC tissue array, as described in Figure 1. (B) SSX1 expression was negatively correlated with KIAA1456 expression in EOC tissue arrays. Scale bars indicate 100 μm in the inserts. The results of SSX1 expression were evaluated by the staining scores. *p < 0.05. P<0.001 in (b) was obtained from Pearson correlation test.

**Figure 6:**
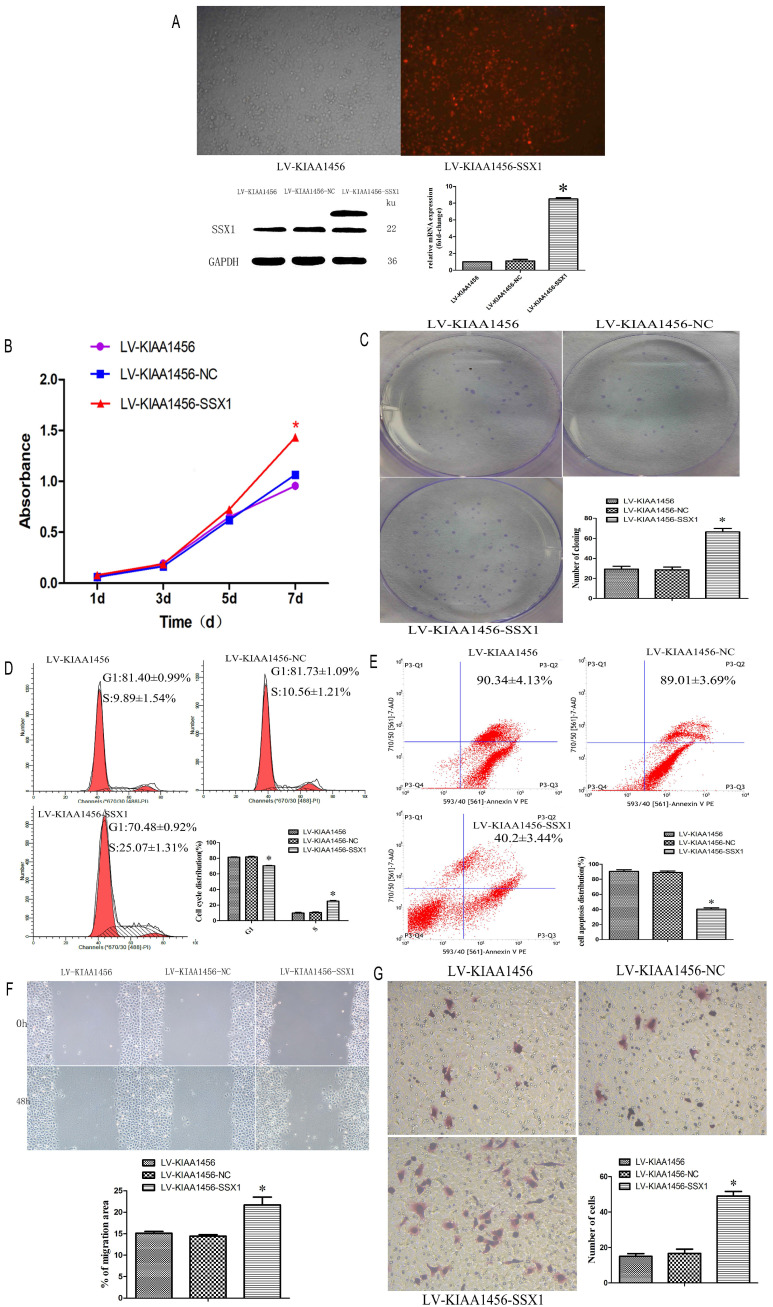
SSX1 rescues the proliferative-suppressive effect of KIAA1456 overexpression in HO8910PM cells. (A) SSX1 was overexpressed in LV-KIAA1456 HO8910PM cells by lentivirus transfection. KIAA1456 protein and mRNA were detected by Western blot analysis and RT-PCR (B and C). Cell viability and proliferation was detected in LV-KIAA1456 and LV-SSX1 cells by CCK-8 and colony formation assays. (D and E) Cell cycle distribution and cellular apoptosis were tested by flow cytometry. (F and G) Wound healing and Transwell assays were performed to assess cell migration and invasiveness. The data was statistically significant at *p < 0.05 as compared to control. Data are represented as mean ± SD of three independent experiments.

Laser confocal analysis was applied to identify the location of SSX1 in the HO8910PM cells transfected with KIAA1456 (Figure 4 A). To determine whether SSX1 is involved in EOC cell proliferation and invasion by KIAA1456, we further estimated the expression of SSX1 in the cells with changed KIAA1456 expression by immunofluorescence and qRT-PCR. The LV-KIAA1456 cells exhibited greatly decreased SSX1 mRNA and protein levels, whereas silencing KIAA1456 in RNAi-KIAA1456 cells dramatically increased SSX1 protein and mRNA levels (Figure 4 B-E). Next, we implemented rescue experiments. EOC cells were cotransfected with over KIAA1456 (LV-KIAA1456) and over SSX1(LV-SSX1) (Figure 6 A), and the ability of LV-KIAA1456 was partially reversed. Cell viability and proliferative ability were evaluated by CCK8 and plate clone formation assay (Figure 6 B and C), Cell cycle distribution was assessed by flow cytometry assay (Figure 6 D and E). Wound healing and Transwell assays were performed to assess cell migration and invasiveness (Figure 6 F and G). These results indicated that KIAA1456 decreased the expression of SSX1, and SSX1 overexpression could partially reverse the KIAA1456-induced biological effect, including the inhibition of cell proliferation, invasion and migration and the induction of apoptosis, thereby attenuating the inhibitory effects of KIAA1456.

### KIAA1456 inhibits AKT signaling pathway in epithelial ovarian cancer cell

**Figure 7:**
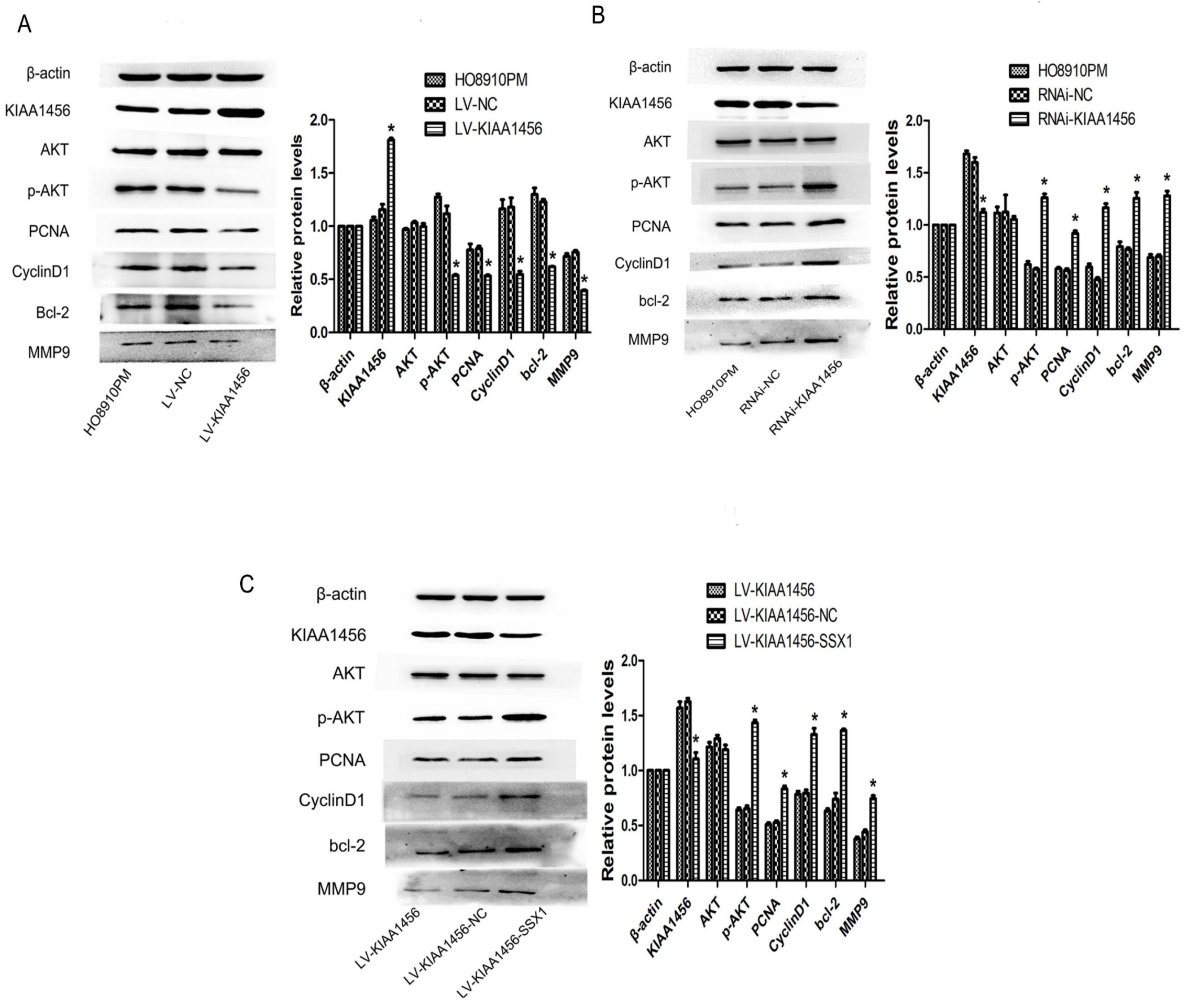
KIAA1456 inhibits the AKT signalling pathway in epithelial ovarian cancer cells. (A-C) Western blot analysis of AKT, p-AKT, PCNA, CyclinD1, MMP9 and Bcl-2 expression in HO8910PM, LV-KIAA1456, RNAi-KIAA1456 and LV-SSX1 cells. (A) KIAA456 overexpression inhibits AKT signalling pathways. (B) Knockdown of KIAA1456 enhances AKT signalling pathways. (C) SSX1 overexpression attenuates the inhibitory effects of KIAA1456 induction on the EOC. The data was statistically significant at *p < 0.05 as compared to control. Data are represented as mean ± SD of three independent experiments.

In our early work, bioinformatics analyses of microarray profiling indicated that the PI3K/AKT signalling pathway was differentially expressed in KIAA1456-overexpressing and control cells (Table SIII). To further investigate the molecular mechanism of KIAA1456 in suppressing the proliferation and invasion of EOC cells, we explored whether this signalling pathway is influenced by KIAA1456 in EOC cells. Western blot analysis was applied to detect its downstream factors related to cell growth and apoptosis. We found that KIAA1456 overexpression reduced the expression of phosphorylated AKT, PCNA and MMP9 and the expression of CyclinD1 and Bcl-2 (Figure 7 A). Consistently, the downregulation of KIAA1456 promoted the expression of p-AKT, PCNA, MMP9, CyclinD1 and Bcl-2 (Figure 7 B). Furthermore, SSX1 overexpression attenuated the inhibitory effects of KIAA1456 induction on the expression of p-AKT, PCNA, MMP9, CyclinD1 and Bcl-2 (Figure 7 C). Hence, these discoveries demonstrated that KIAA1456 can suppress AKT signalling cascades, probably explaining its negative effects on cell proliferation, invasion and migration and positive effects on apoptosis.

## Discussion

The treatment of ovarian tumours remains a huge clinical challenge because of late diagnosis and limited effective treatment options for advanced metastatic carcinoma 25. Genetic susceptibility, environmental factors and viral infections play crucial roles in ovarian tumour etiopathogenesis; however, the specific molecular mechanism for the genesis and development of tumours remains indistinct 26-28. KIAA1456 can catalyse the swing of tRNA bases for tRNA maturation, which plays an important role in the occurrence and development of tumours. KIAA1456 can be used as a tumour suppressor gene in lung cancer, inhibiting the proliferation, migration and invasion of lung cancer cells 11. KIAA1456 is a tumour suppressor located at chromosome position 8p22, a phosphoprotein with phosphorylation that plays an important role in tumour inhibition behaviours 7. No study has investigated the involvement of the KIAA1456 gene in ovarian cancer. We confirmed that KIAA1456 inhibited EOC cell proliferation and metastasis in our previous study. KIAA1456 in many other tumour tissues was lower in expression compared with paired normal tissues in respect to the promoter methylation of KIAA1456, which was in accordance with previous findings 7. These arguments suggest the viewpoint that KIAA1456 acts as a novel tumour suppressor gene. To elucidate the role of KIAA1456 in EOC progression, in the current study, we investigated correlations between KIAA1456 expression levels and clinical pathological characteristics in EOC. Compared with normal ovarian tissues, EOC tissues frequently showed lower KIAA1456 expression, as shown by the immunohistochemical staining. Meanwhile, KIAA1456 expression in malignant EOC cells was significantly lower than in benign ovarian tumor cells. Furthermore, we found that the expression levels of KIAA1456 were lower in high-grade EOCs compared with low-grade EOCs, indicating that the expression level of KIAA1456 was inversely associated with the pathological grade of EOC. In addition, low KIAA1456 expression was closely related to poor overall survival in patients with ovarian carcinoma.

Some striking characteristics of malignancies are unrestricted multiplication and resistance to apoptosis. Abnormalities in cell apoptosis and cell cycle also result in unusual tumour cell multiplication 25-28. To further research the biological functions of KIA1456 in epithelial ovarian tumours, we explored the effect of KIAA1456 overexpression and down-expression on cell proliferation and metastasis in HO8910PM cells. Our results revealed that the abnormal overexpression of KIAA1456 in HO8910PM cells could remarkably suppress cell proliferation, migration and invasion and induce G1/S arrest and the apoptosis of EOC cells. By contrast, the knockdown of KIAA1456 induces the opposite reaction. *In vivo* tumourigenicity experiments verified that KIAA1456 could restrain ovarian cancer proliferation. These consequences were consistent with the reported roles of KIAA1456 on other tumour cells. Accordingly, there may be a promote relationship to the proliferation, migration, invasion and resistance relationship to apoptosis in EOC cells while the expression of KIAA1456 decreased. The precise mechanism of KIAA1456 in regulating the multiplication and metastasis of EOC cells remains unclear.

Furthermore, the mechanisms underlying how KIAA1456 influences EOC progression are unclear. SSX1 belongs to a family of highly homologous proteins in the group of SSX antigens, which exhibit a specific translocation between chromosome X and chromosome 18 t(X;18) (p11.2; q11.2) 17. SSX1 protein has a distinct DNA binding domain and is localised in the nucleus and associated with transcriptional regulation 16. In respects, SSX1 is an excellent candidate target of cancer immunotherapy, as it is frequently expressed in cancer cells. In our early work, we performed microarray profiling to identify putative targets of KIAA1456 that might be involved in ovarian cancer suppression and found that SSX1 is the most strongly downregulated gene in KIAA1456-overexpressing cells. SSX1, as a key downstream transcription factor, plays a critical role in cell proliferation and metastasis. Little is known whether SSX1 is involved in KIAA1456's regulation of proliferation and apoptosis in epithelial ovarian cancer cells. In this study, we found that SSX1 was highly expressed in malignant EOC tissues, in contrast with benign epithelial ovarian tumour tissues and normal ovarian tissues. Pearson's correlation analysis showed that KIAA1456 was negatively correlated with SSX1 expression in EOC tissues. Furthermore, the partial KIAA1456-induced biological effects on EOC cells, including cell growth inhibition, migration, invasion and apoptosis induction, could be reversed by the overexpression of SSX1.

The PI3K/AKT pathway plays an important role in various cell functions in EOC 29. Therefore, we examined whether KIAA1456 can regulate the expression of the AKT signalling pathway. We found that KIAA1456 overexpression in EOC cells could inhibit the phosphorylation of AKT; conversely, the downregulation of KIAA1456 could increase the phosphorylation of AKT, demonstrating that KIAA1456 can inhibit cell proliferation and promote apoptosis, partly via the AKT signalling pathway. In addition, AKT may be connected with cancer proliferation and apoptosis in epithelial ovarian tumour cells, possibly because of the involvement of AKT in the regulation of downstream signalling molecules, including CyclinD1, Bcl2, PCNA and MMP9. Moreover, we found that KIAA1456 overexpression suppressed cell growth and promoted apoptosis through the downregulation of p-AKT, CyclinD1, PNCA, MMP9 and Bcl-2 expression. These findings suggest that KIAA1456 can suppress EOC cell proliferation, invasion and promote apoptosis, partly through the AKT signalling pathway.

Our observations showed that KIAA1456 overexpression in aggressive EOC cells with low KIAA1456 levels dramatically blocked tumour growth, invasion and migration* in vivo*. Considering that KIAA1456 is lowly expressed in EOC was correlated with pathological grade. Simultaneously, the present study provides evidence that KIAA1456 as a tumour suppressor gene could suppress proliferation and metastasis of EOC cells by inhibiting the SSX1 and AKT signalling pathways. Our findings indicate that KIAA1456 is a novel tumour suppressor gene and may be a possible therapeutic target in clinical practice.

## Supplementary Material

Supplementary tables.
