# Supplementary material for: The inhibitory effect of KIAA1456 on the proliferation and metastasis of epithelial ovarian cancer through SSX1 and AKT signaling pathway
Source: J Cancer. 2023 Mar 27;14(5):770–83. doi: 10.7150/jca.81587 (PMC10088888; doi:10.7150/jca.81587)
Supplement: Supplementary file 1 — Supplementary tables. [file jcav14p0770s1.pdf]

Table 1

Correlation expression of KIAA1456 in ovarian tissues， benign and malignant human epithelial ovarian cancer

Variable	n	KIAA1456 expression					P
		< 10%	10-20%	20-30%	> 30%	Median positive (%)	
Tissue							0.000
Normal ovarian tissue	15	0	0	5	10	29.48	
Benign EOC	15	0	6	9	0	18.72	
Malignant EOC	45	23	21	1	0	8.91	
Histologic grade							0.000
Low-grade	15	3	11	1	0	12.09	
High-grade	30	20	10	0	0	6.04	
Age（years）							0.9414
≤50	37	9	14	10	4		
> 50	38	14	13	8	3		

Table 2 Microarray analysis identified a list of genes significantly differentially expressed after KIAA1456 overexpression.

**Gene Symbol**	**Accession Number**	**Gene Description**	**Fold Change**	**p-value**	**Gene Feature**
IQGAP1	NM_003870	"Homo sapiens IQ motif containing GTPase activating protein 1 (IQGAP1), mRNA."	1.702313	0.000584	up
UBE4A	NM_001204077	"Homo sapiens ubiquitination factor E4A (UBE4A), transcript variant 2, mRNA."	1.419223	0.00054	up
PSMD1	NM_001191037	"Homo sapiens proteasome (prosome, macropain) 26S subunit, non-ATPase, 1 (PSMD1), transcript variant 2, mRNA."	1.365103	0.000545	up
KIAA1456	NM_001099677	"Homo sapiens KIAA1456 (KIAA1456), transcript variant 2, mRNA."	1.314254	0.000115	up
SMARCA1	NM_003069	"Homo sapiens SWI/SNF related, matrix associated, actin dependent regulator of chromatin, subfamily a, member 1 (SMARCA1), transcript variant 1, mRNA."	1.286039	0.000653	up
TRIM29	NM_012101	"Homo sapiens tripartite motif containing 29 (TRIM29), mRNA."	1.222872	0.000775	up
SSX1	NM_005635	"Homo sapiens synovial sarcoma, X breakpoint 1 (SSX1), mRNA."	5.73389	5.10E-05	down
CYP1A1	NM_000499	"Homo sapiens cytochrome P450, family 1, subfamily A, polypeptide 1 (CYP1A1), mRNA."	-2.26197	0.013938	down
IGHD2-21	ENST00000390572	cdna:known chromosome:GRCh37:14:106354409:106354436:-1 gene:ENSG00000211912 gene_biotype:IG_D_gene transcript_biotype:IG_D_gene	-1.5694	0.026841	down
IER3	NM_003897	"Homo sapiens immediate early response 3 (IER3), mRNA."	-1.50082	0.020279	down
HMOX1	NM_002133	"Homo sapiens heme oxygenase (decycling) 1 (HMOX1), mRNA."	-1.47563	0.003978	down

Table 3 KEGG pathway analysis of differentially expressed genes in KIAA1456-overexpressing and control cells.

Pathway	Diff Gene Counts in Pathway	Enrichment Score	P-value	FDR	Gene Symbols
RNA transport	16	13.41944	1.95E-13	3.23E-11	|EIF2B2|EIF3A|GEMIN5|…
Protein processing in endoplasmic reticulum	12	9.944049	7.95E-09	6.56E-07	|SEC24A|CANX|UBE2G1|…
Lysosome	9	10.20895	5.72E-07	3.15E-05	|CTSL1|AP3M1|DNASE2|…
Proteoglycans in cancer	11	6.706027	1.91E-06	7.89E-05	|ITGAV|FN1|IQGAP1|…
Pathways in cancer	12	5.078459	1.11E-05	0.000366	|MET|HSP90AA1|ITGAV|…
Proteasome	5	15.72591	3.29E-05	0.000905	|PSMB7|PSME2|PSMD1|…
Antigen processing and presentation	6	10.12595	6.00E-05	0.001413	|HSPA4|CTSB|HSP90AA1|…
Purine metabolism	7	5.599515	0.000564	0.011625	|PRPS2|POLR2B|PNP|…
Ubiquitin mediated proteolysis	6	6.01687	0.00105	0.01575	|UBE4A|HERC3|UBE3A|…
Metabolic pathways	20	2.327805	0.001258	0.0173	|AMD1|PNP|EPRS|…
PI3K-Akt signaling pathway	8	3.190502	0.007973	0.058966	|ITGB1|MET|ITGAV|…
